# Prothrombotic Phenotype in COVID-19: Focus on Platelets

**DOI:** 10.3390/ijms222413638

**Published:** 2021-12-20

**Authors:** Cristina Barale, Elena Melchionda, Alessandro Morotti, Isabella Russo

**Affiliations:** Department of Clinical and Biological Sciences, Turin University, 10043 Orbassano, Turin, Italy; cristina.barale@unito.it (C.B.); e.melchionda@gmail.com (E.M.); alessandro.morotti@unito.it (A.M.)

**Keywords:** platelet activation, COVID-19, SARS-CoV-2, thrombosis, inflammation, immunothrombosis

## Abstract

COVID-19 infection is associated with a broad spectrum of presentations, but alveolar capillary microthrombi have been described as a common finding in COVID-19 patients, appearing as a consequence of a severe endothelial injury with endothelial cell membrane disruption. These observations clearly point to the identification of a COVID-19-associated coagulopathy, which may contribute to thrombosis, multi-organ damage, and cause of severity and fatality. One significant finding that emerges in prothrombotic abnormalities observed in COVID-19 patients is that the coagulation alterations are mainly mediated by the activation of platelets and intrinsically related to viral-mediated endothelial inflammation. Beyond the well-known role in hemostasis, the ability of platelets to also release various potent cytokines and chemokines has elevated these small cells from simple cell fragments to crucial modulators in the blood, including their inflammatory functions, that have a large influence on the immune response during infectious disease. Indeed, platelets are involved in the pathogenesis of acute lung injury also by promoting NET formation and affecting vascular permeability. Specifically, the deposition by activated platelets of the chemokine platelet factor 4 at sites of inflammation promotes adhesion of neutrophils on endothelial cells and thrombogenesis, and it seems deeply involved in the phenomenon of vaccine-induced thrombocytopenia and thrombosis. Importantly, the hyperactivated platelet phenotype along with evidence of cytokine storm, high levels of P-selectin, D-dimer, and, on the other hand, decreased levels of fibrinogen, von Willebrand factor, and thrombocytopenia may be considered suitable biomarkers that distinguish the late stage of COVID-19 progression in critically ill patients.

## 1. Introduction

Despite coronavirus diseases 2019 (COVID-19) infection being associated with a broad spectrum of presentations, a common finding in COVID-19 patients and appearing as a consequence of severe endothelial injury with endothelial cell membrane disruption is the presence of alveolar capillary microthrombi [1]. These observations clearly point to the identification of a COVID-19-associated coagulopathy, which may contribute to thrombosis, multi-organ damage, and because of severity and fatality.

Given the knowledge we have, there is still no clarity on the pathophysiological mechanisms involved in arterial and venous thrombosis during COVID-19 disease and, particularly, some aspects of platelet susceptibility to severe acute respiratory syndrome coronavirus 2 (SARS-CoV-2) are in contrast. However, the literature agrees on the occurrence of prothrombotic abnormalities and, in this scenario, platelets have been shown to be deeply involved. Traditionally platelets are recognized as key actors in hemostasis. Indeed, we have to consider that platelets are also deeply implicated in the host defense in case of infections [2] given that, together with other immune cells and coagulation process, they act as modulators and effectors of immune cells other than clot formation [3]. In vertebrates, platelets are able to respond to pathogens mainly by promoting neutrophil extracellular traps (NETs) release and indirectly disposing of them [4]. The ability to prime macrophages, recruit and activate neutrophils, and participate actively to intravascular thrombosis places platelets as a link between host defense and thromboinflammation. Indeed, inflammation may cause hemostatic alterations leading to thrombosis [5,6] and, on the other hand, thrombosis may exacerbate inflammation [7], thus promoting a loop which increases tissue damage and thrombotic complications [8,9].

In the pathogenesis of COVID-19, the excessive release of cytokines is crucial in further influencing the systemic hemodynamic aberrations and cardiovascular diseases (CVD) and, in this context, platelets secrete a number of pro-inflammatory cytokines, chemokines, and growth factors that significantly contribute to thromboinflammation [10], which is responsible for infection sequelae and severity in organ failure.

Of course, in the crosstalk between inflammation and thrombosis, platelets are required to talk with other circulating cells, such as monocytes and macrophages, and each of them talks with endothelial cells [11,12]. As documented by reports in patients deceased due to the fact of COVID-19, an extensive platelet–fibrin clot formation in the pulmonary microvasculature was found in 80–100% of lungs and in other organs examined [13] and marked inflammation has been established to be a typical clinicopathological feature that worsens COVID-19 prognosis [7,14,15].

Circulating platelet–neutrophil, –monocyte and –T-cell aggregates were found to be significantly increased in patients with COVID-19 in comparison with healthy subjects [16], and platelets themselves manifest hyperreactivity [16,17], thus contributing to COVID-19 pathophysiology.

This review will focus on the link between the prothrombotic status of patients suffering from COVID-19 and platelet activation, thus providing an overview of the inherent links between thrombosis and the immune response, which is useful for understanding how these relationships may promote the prothrombotic consequences due to the presence of SARS-CoV-2 infection.

## 2. Peculiar Aspects of Thrombotic Events in COVID-19

Although the exhaustive pathobiology profile of thrombosis due to the fact of COVID-19 has not yet been fully clarified, one significant finding that emerges in the difference between COVID-19 and non-COVID-19 disorders is that the coagulation alterations are mainly mediated by the activation of platelets and intrinsically related to viral-mediated endothelial inflammation [18].

From a histopathological point of view, SARS-CoV-2 distinguishes from other viruses that share the tropism for the respiratory tract. Lungs from patients who died due to the fact of COVID-19 showed widespread thrombosis with microangiopathy combined with a severe endothelial injury with virus infiltration, disrupted cell membrane, a dense perivascular infiltration of T lymphocytes, and an aberrant condition of macrophage activation [1].

These and other distinctive histopathological features, such as the uncontrolled inflammatory response and endothelial cell apoptosis, confer to COVID-19 characteristics not comparable to any other equally severe viral respiratory infections [1]. COVID-19 patients who develop thrombotic complications show hypercoagulability combined with increased levels of coagulation factors, acquired antiphospholipid antibodies, and reduced production of endogenous anticoagulant molecules [18].

As mentioned, patients affected by COVID-19 experience a range of diseases from mild illness to severe multiple organ dysfunction having as notable manifestation the fatal hypoxemic respiratory failure. Nevertheless, the non-pulmonary organ damages associated with a platelet prothrombotic phenotype have emerged as important predictors of mortality [19] given that several immunomodulatory molecules regulating leukocyte and endothelial function are released by platelets making them a major player in the thrombo-inflammation complications of COVID-19 [20,21].

## 3. Platelet Activation

**Figure 1:**
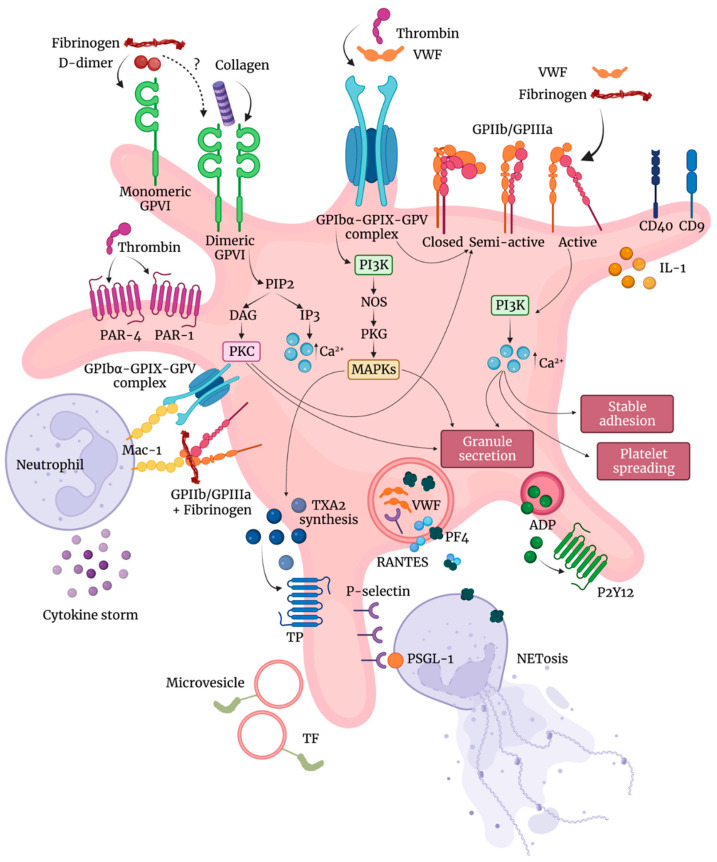
Platelet activation in COVID-19. Platelets from COVID-19 patients show higher expression of the GPIbα–GPIX–GPV complex, GPIIb/GPIIIa complex, CD9, and CD40. Activated platelets also show increased secretion of IL-1 and release of microvesicles–TF (tissue factor) complexes. While fibrinogen and D-dimer bind to monomeric GPVI, collagen binds to dimeric GPVI, leading to the activation of PKC (protein kinase C). Thrombin and VWF (von Willebrand factor) bind to the GPIbα–GPIX–GPV complex, leading to the activation of PI3K/MAPKs and to a conformational change of the GPIIb/GPIIIa complex in its active form, able to bind fibrinogen and VWF and to activate PI3K, leading to an increase in the intracellular Ca^2+^. These pathways lead to platelet adhesion, spreading, TXA2 (thromboxane A2) synthesis, and granule secretion of ADP, PF4 (platelet factor 4), VWF, RANTES, and P-selectin. PF4–RANTES heterodimerization and the binding of P-selectin to PSGL-1 (P-selectin glycoprotein ligand 1) stimulate platelet–neutrophil interaction and promote NETs (neutrophil extracellular traps) formation. The binding of GPIbα–GPIX–GPV and GPIIb/GPIIIa÷fibrinogen complexes to Mac-1 (macrophage antigen-1) regulates platelet–leukocyte interaction exacerbating the inflammatory response.

Platelets, small anucleated cells released by megakaryocytes [22,23], are known to influence not only vascular hemostasis but also immune response, tumor progression, and other inflammatory processes [24]. Specifically, during sepsis, platelets can promote endothelial dysfunction, NETs formation, and generation of microthrombi, thus exacerbating coagulation and inflammation. Moreover, the ability of antiplatelet therapy during sepsis to reduce the uncontrolled inflammation, coagulation, and damage to organ function and improve prognosis of patients has confirmed the role of platelet hyperactivation in promoting the interaction of platelets–inflammatory cells–endothelial cells with the subsequent cascade reaction between inflammation and coagulation [25,26,27,28]. Platelets contain three types of granules (i.e., alpha, dense, lysosomes) and their activation results in the secretion of molecules stored in these granules able to modulate aggregation and thrombus formation [29] (Figure 1).

Soluble fibrinogen binding to platelet integrins activates platelets and promotes clot formation [29,30,31]. One of the more important platelet integrins to which fibrinogen binds is the integrin αIIbβ3 [32] that is involved in platelet spreading [33]. In resting platelets, integrin αIIbβ3 is usually inactive with a low affinity for ligands. After platelet stimulation, the conformation of the integrin αIIbβ3 changes resulting in a receptor with a higher affinity state for ligands and allowing further signaling events [32]. Glycoprotein (GP) VI is the main platelet receptor for collagen, exposed in the case of endothelial injury [34,35]. Dimerization of GPVI is required for collagen binding [36], while [37] only monomeric GPVI is able to bind fibrin (ogen) and D-dimer [38,39].

The activation of more relevant fibrin (ogen)- and D-dimer-induced signaling pathways involved in platelet activation [29,31,40] observed in COVID-19 are illustrated in Figure 1. Activation of these signaling pathways triggers aggregation and activation of platelets combined with conformationally shape change and the subsequent clot formation and eventually clot retraction [41,42].

In comparison with controls, platelets from hospitalized stable COVID-19 patients show enhanced levels of the platelet activation markers P-selectin and lysosomal-associated membrane protein 3 (LAMP-3), and significantly higher expression of the transmembrane integrins GPIIb/GPIIIa complex, GPIbα, GPIX, CD9, and CD40 [43]. In the same study, the authors show that after stimulation with thrombin receptor activating peptide (TRAP), platelets respond with higher expression of the collagen receptor GPVI [43].

The expression of P-Selectin, a 120 kDA transmembrane protein, on the platelet surface subsequent to platelet activation regulates neutrophil–platelet, platelet–platelet, and monocyte–platelet interactions, becoming a driver for activation of neutrophil integrins, formation of NETs [44], and tissue factor expression [45]. The upregulation of the integrins GPIIb (CD41) and GPIIIa (CD61) and the von Willebrand factor (VWF) receptor subunits, GPIbα and GPIX, known to regulate platelet–leukocyte interactions, together with P-selectin may contribute to exacerbate the inflammatory response in COVID-19 patients [44,46,47].

On the effectiveness of antiplatelet therapy in COVID-19 patients, both positive and negative results have been found. In a retrospective, observational study, including 412 hospitalized patients with COVID-19, aspirin administration reduced the need of mechanical ventilation (aspirin group: 35.7% vs. non-aspirin group: 48.4%, *p* = 0.03) even if the mortality did not differ (aspirin group: 26.5% vs. non-aspirin group: 23.2%, *p* = 0.51 [48]).

In a randomized, controlled, open-label, platform trial having the primary outcome at 28 day mortality, aspirin was not associated with reduced mortality or risk of progressing to invasive mechanical ventilation. Nevertheless, in the same study a subtle increase in the likelihood of being discharged alive within 28 days was observed, even if not statistically significant (RR: 0.96, 95% CI: 0.89–1.04) [49].

## 4. Platelets and Immunothrombosis

SARS-CoV-2 infection induces immunothrombosis, a process in which the interaction between activated neutrophils, monocytes, coagulation cascade, and platelets leads to intravascular clot formation from small to large vessels [50].

Beyond the well-known role in hemostasis, the ability of platelets to release also various potent cytokines and chemokines has elevated these small cells from simple cell fragments to crucial modulators in the blood, including their inflammatory functions, which have a large influence on the immune response during infectious diseases [51,52]. Platelets express several immunoreceptors making them sentinels ready to recognize intravascular pathogens. In order to ensure pathogen clearance, platelets activate immune cells even if platelets themselves can directly limit pathogen growth through the release of antimicrobial molecules. However, a condition of aberrant platelet activation leads to inflammation and thrombotic complications.

In inflammatory conditions, platelet–neutrophil interaction promotes further recruitment of neutrophils into sites of inflammation [53]. The P-selectin glycoprotein ligand 1 (PSGL-1), expressed on neutrophils and binding to platelet P-selectin, mediates neutrophil–platelet interaction [54]. Blocking the P-selectin-mediated platelet interaction with neutrophils by using antagonists to P-selectin and GPIIb/IIIa or reducing circulating platelets have been shown to significantly reduce recruitment of neutrophils and vascular permeability, improve gas exchange, and prolong survival in sepsis-induced models of acute lung injury [55,56].

Neutrophil adhesion to platelets is reinforced by the β_2_-integrin macrophage antigen-1 (Mac-1, CD11b/CD18) binding to GPIbα on the platelet surface and the simultaneous binding of fibrinogen GPIIb/GPIIIa on platelets and CD11b/CD18 on neutrophils [57,58] (Figure 1).

Furthermore, the deposition by activated platelets of the chemokine platelet factor 4 (PF4; CXC chemokine ligand 4 [CXCL4]) [59] at sites of inflammation promotes adhesion of neutrophils on endothelial cells [60,61] and thrombogenesis [62,63]. Indeed, platelets are involved in the pathogenesis of acute lung injury also by promoting NETs formation and affecting vascular permeability [64]. Mechanistically, PF4 has been reported to be a crucial mediator in forming NETs [62]. PF4 heterodimerization with regulated upon activation of normal T cell expressed and presumably secreted (RANTES), another alpha-granule stored chemokine released upon platelet stimulation, induces activation and recruitment of inflammatory cells [61,65]. Conversely, the disruption of PF4–RANTES interaction reduces platelet–neutrophil aggregates and inhibits NETs formation, neutrophil recruitment, and reduces vascular permeability [62]. These findings support the concept that the crosstalk among integrins may be critical in synergizing platelet effects on modulating inflammatory response.

Indeed, as already mentioned, the inhibition of platelet–neutrophil interaction by blocking P-selectin or GPIIb/IIIa [55,66] also reduces the severity of acute lung injury, thus suggesting that the platelet-mediated infiltration of neutrophils is a basic mechanism in different inflammatory settings [55,66,67] even if factors influencing leucocyte emigration in the microcirculation of inflamed lung are also others [68].

PF4 is a glycosaminoglycan-binding protein able to neutralize the anticoagulant properties of antithrombin towards thrombin and the coagulation factor Xa [69,70,71,72].

Upon platelet stimulation, the tetramer PF4 transfers to polysaccharides, such as heparin, more highly sulfated than its proteoglycan carrier [73,74,75]. Taking in mind the important role of heparan sulfate proteoglycans in providing suitable storage sites at vascular wall for coagulation inhibitors, such as antithrombin, specifically at level of endothelium and the high PF4 binding affinity [76], we can understand the importance of PF4 to negatively interfere with the anti-thrombotic heparin-like activity of endothelium [72,77].

**Figure 2:**
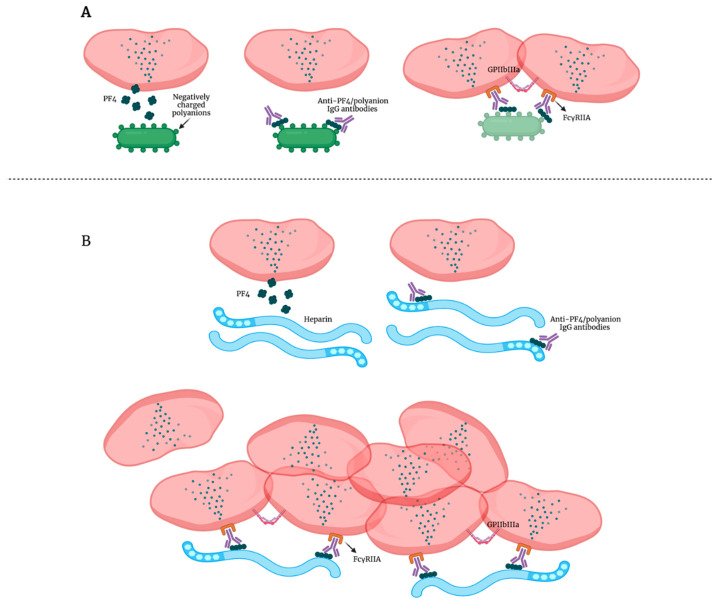
(**A**) PF4 role in bacterial killing. PF4 (platelet factor 4) stored in alpha-granules is released. When PF4 binds negatively charged polyanions, it undergoes a conformational change. This binding leads production of anti-PF4/polyanion IgG antibodies. The IgG–PF4 complex binds the FcγRIIA receptor on the platelet surface and promotes bacterial killing. (**B**) PF4 role in HIT. PF4 binds negatively charged molecules like heparin leading to the formation of anti-PF4/polyanion IgG antibodies. The IgG–PF4 complexes induce HIT (heparin-induced thrombocytopenia), an immune complication of heparin therapy clinically associated with thrombocytopenia and thromboembolic complications.

When the positively charged PF4 binds negatively charged polyanions, a conformational change of PF4 occurs with the consequent exposure of antigenic neoepitopes [78,79] (Figure 2A).

These neoepitopes cause production of anti-PF4/polyanion IgG antibodies that, in the case of bacterial infection, represent a mechanism by which platelets directly (Figure 2A) [80] or indirectly promote bacterial phagocytosis [81,82], thus killing the opsonized anti-PF4/polyanion IgG bacteria. In this way, a general mechanism of recognition allows targeting of a wide spectrum of invading pathogens with a single clonal antibody and platelets with their chemokine PF4 and Fc-gamma receptor (FcγR)IIA are shown as a bridge between innate and acquired immunity.

Taking in mind that the platelet IgG Fc-domain is a strong cell activator, the immune receptor FcγRIIA IgG-PF4 binding to platelet FcγRIIA leads to a consistent cell stimulation including platelet adhesion, aggregation, release of both alpha- and dense granule content, thromboxane A2 production and cytoskeleton rearrangement [83,84,85].

It has been demonstrated that platelet FcγRIIA is dynamically linked to GPIIb/GPIIIa on the platelet membrane; thus, the fibrinogen secreted after platelet stimulation further supports platelet interaction and spreading [83].

However, besides cooperating in host defense from pathogens, the anti-PF4/polyanion antibodies can also induce the so-called heparin-induced thrombocytopenia (HIT) without prior heparin exposure, a response known as spontaneous HIT [86] (Figure 2B)*,* an immune complication of heparin therapy clinically associated with thrombocytopenia and life-threatening thromboembolic complications with mortality which exceeds 20% [87].

Of note, there has also been reported crosstalk among the complement system, which is constituted of more than 30 types of soluble plasma proteins or membrane proteins and platelets that may exacerbate the hypercoagulable condition in COVID-19. Actually, C3a and C5a fragments are known to stimulate mast cell degranulation and endothelial cell activation, thus promoting prothrombotic events mainly by stimulating TF and VWF secretion, respectively [88]. Furthermore, C3a fragment, in particular, directly stimulates platelets, suggesting that the over-activation of the complement system in SARS-CoV-2-infected patients may contribute to exacerbating the risk of thrombosis [89] not only for the accumulation of inflammatory infiltrates in the pulmonary alveoli but also for leading to a fatal hypercoagulable state This could support a role for complement inhibition as potential therapeutic approach to treat patients SARS-CoV-2 infected [90].

## 5. Platelets and Inflammation

Indeed, at the beginning it was not so clear how the inflammatory response to SARS-CoV-2 was intertwined with coagulation disorders. Then, evidence from both translational and basic research studies has better clarified the close relationship among innate immunity, platelets, and coagulation factors, not only in capturing and fighting invading pathogens but also in contributing to tissue injury due to the excessive inflammatory response. Consistently, Manne et al. [16] showed in COVID-19 patients both higher surface P-selectin expression and higher circulating platelet–leukocyte aggregates and Nicolai et al. reinforced the concept that immunothrombosis is the link between multiorgan failure and thrombotic events [91]. Surface activation markers of platelets and neutrophils as well as functional assays showed marked histopathological changes in vessel microcirculation. These abnormalities included microvascular thrombotic formations containing neutrophils, platelets, and NETs, now considered hallmarks of SARS-CoV-2 infection [91]. Furthermore, the same authors in in vitro assays found that platelets and neutrophils from infected patients, in comparison with those from healthy subjects, exhibited abnormal activation in the circulation [91].

In the presence of bacterial or viral infections, the immune system reacts through the release of both proinflammatory and anti-inflammatory cytokines essential to control and eliminate pathogens. However, following exposure to cells presenting SARS-CoV-2 antigen, T-cell reactivity has been shown to amplify cytokine release (cytokine storm) resulting in a positive feedback between immune cells and cytokines [92,93]. Indeed, in an infectious clinical setting, platelets express a number of immunoreceptors that enable them to act as specialized sentinels able to sense and, via receptors, recognize pathogens from all major classes of microorganisms invading the bloodstream [94]. Given that their primary function is constantly to scan the endothelium to detect the presence of any vessel damage, platelets can act as first responders to invading pathogens resulting in platelet activation and release of antimicrobial molecules, cytokines and adhesion molecules, that regulate the host immune cell response against infection [51]. However, the presence of aberrant platelet activation can promote inflammation and thrombosis that are not two independent and separate processes being connected by points making them an integral part of the defensive host response [8].

Actually, platelets from patients with COVID-19 showed a hyperreactive phenotype [20,43] and upregulation in the release of soluble immunomodulatory factors [21], indicating the involvement of platelets in the pathogenesis of thromboinflammation in this viral infection.

In addition, the genomic dsRNA virus can modulate interferon secretion and activate nuclear factor kappa-light-chain-enhancer of an activated B cells (NF-kB) signaling pathway leading to release of huge amounts of interferon type 1 and proinflammatory cytokines [95]. The cytokine storm described in COVID-19 can induce activation of platelets that release microvesicles with procoagulant activity and express tissue factor (TF) on their surface [96,97] and the presence of cytokine storm [98,99] with the consequent hyperactivation of platelets may become responsible for CV complications [3]. Actually, coagulation disorders are known to be linked to viral infections [100] and an increased incidence of CV events has been observed after influenza infection [101]. Furthermore, coagulopathies including thrombocytosis, disseminated intravascular coagulation (DIC), and thromboembolism were also observed in SARS-CoV-1 [102,103].

## 6. SARS-CoV-2 Effects on Platelets

Blood from patients with COVID-19 contains SARS-CoV-2, is infectious [104,105,106], and high mRNA levels of SARS-CoV-2 correlate with severity of infection [106,107].

In viral infections, such as influenza, human immunodeficiency, and hepatitis C, platelets internalize virions, resulting in platelet activation [52,108]. A direct action of SARS-CoV-2 on platelets is still controversial. In a study by Barrett et al., SARS-CoV-2 viral particles were found in megakaryocytes morphologically active in platelet production in the bone marrow [17] and within lung in deceased COVID-19 patients [17]. Consistently, viral particles were also found in platelets in approximately 39% of circulating platelets not showing changes in their morphology. Obviously, this suggests that SARS-CoV-2 is either transferred from megakaryocytes to platelets or directly engulfed by circulating platelets.

The mechanism by which megakaryocytes and platelets take up SARS-CoV-2 has been explored and platelets have been reported to express the receptor for SARS-CoV-2 angiotensin-converting enzyme (ACE) 2 [105,106]. It is known that SARS-CoV-2 binds to and enters through cells that express ACE2 [109] and promotes an immediate downregulation of this receptor [110]. Virus binding to ACE2 receptor determines an increase of its substrate Angiotensin II (Ang II) with an impact on immune, vascular endothelial and coagulation responses [111]. Actually, as a result of Ang II accumulation secondary to ACE2 downregulation, clot formation may also occur for the consequent overexpression of TF [112], a transmembrane protein serving as a high affinity receptor and cofactor for coagulation factors VII and VIIa [113,114,115].

TF expression is negligible in healthy non-inflamed endothelial cells, but it can be induced by a number of proinflammatory stimuli including viruses [116,117,118].

TF binding to factor VII initiates the extrinsic coagulation cascade with generation of factor Xa and thrombin [115] which, in turn, activates platelets and triggers the conversion of fibrinogen to fibrin, essential for blood clotting [119]. COVID-19 patients show elevated TF activity in circulating extracellular vesicles associated with disease severity and mortality [120]. Pulmonary histopathology studies with the characterization of CD61+ platelet thrombi in COVID-19 patients with ARDS showed that CD61+ areas were higher in COVID-19 vs. non-COVID-19 ARDS samples [121]. Interestingly, the same authors found that higher levels of fibrin and activated platelets in PF4-positive thrombi correlated to high TF protein expression throughout lung tissue samples in which both arterial and venous thrombi and microangiopathy were observed [121].

Traces of SARS-CoV-2 mRNA, detected by reverse transcription quantitative real-time PCR (RT-qPCR), were found in isolated platelets in some studies [16] but not in all [122]. Furthermore, it has been found that SARS-CoV-2 mRNA can entry platelets also through mechanisms independent of ACE2 receptor [16].

In any case, platelet hyperactivation in COVID-19 patients has been documented by several studies [16,21,106,123,124] and increased aggregation, alpha-granule secretion, and thrombus formation seem to be also induced by a spike (S) protein fragment binding to platelets [105]. Indeed, SARS-CoV-2 S protein is formed by protruding homotrimers that play a key role in virus attachment to ACE2 receptor of target cells [125]. However, receptor binding per se could not explain each coagulopathy observed in patients affected by COVID-19. Actually, it has been found that S protein can be shed and free S protein subunits were detected in different organs and urine [126]. The coronavirus S glycoprotein is a class I viral fusion protein formed by S1 and S2 subunits [127]. The subunit S1 mediates receptor binding [128], while S2 is responsible for virus–cell membrane fusion [129]. In COVID-19 patients, free S1 particles have been detected in circulation and seem to be involved in the pathogenesis of the disease [130]. Interestingly, a confirmation of SARS-CoV-2 effects on platelets comes from a study carried out by Grobbelaar et al., who showed the in vitro ability of S protein to directly interact with platelets and fibrinogen to cause blood hypercoagulation [126]. Specifically, after that whole blood samples from healthy subjects were exposed to isolated SARS-CoV-2 S protein S1 subunit, platelet hyperactivation and major ultrastructural changes were noted.

The first communication between hemostasis and inflammation occurs at the level of endothelium. Under physiological conditions, the balance between pro- and anti-thrombotic factors released by endothelial cells preserves an intact endothelium; conversely, if endothelium is damaged, it loses its anti-inflammatory and anti-thrombotic properties becoming suitable for inflammatory and prothrombotic environment [8,11,12,131]. While platelets physiologically contribute to guarantee the integrity of basal barrier of the alveolar capillaries, they may also play an important role in lung injury in a variety of pulmonary disorders [132]. The involvement of platelet–leukocyte aggregates and platelet–endothelial interactions in the pathogenesis of acute lung injury has already been observed [133].

As already mentioned, during SARS-CoV-2 infection, lung tissue injury and damage of lung endothelial cells promote platelet aggregation with the formation of microthrombi and consequent consumption of platelets [134]. A number of circulating and dysregulated coagulation and inflammation biomarkers, including D-dimer, P-selectin, fibrinogen, and VWF and various cytokines, can directly bind to endothelial cell receptors thus influencing signaling pathways involved in endotheliopathy [1,135]. Certainly, it has to be considered that platelet hyperactivation may be also the consequence of a damaged endothelium as well as of the cytokine storm occurring during SARS-CoV-2 infection [136]. Actually, alterations in endothelial cell functions cause the decreased production of molecules, such as nitric oxide and prostacyclin, known to prevent platelet adhesion and the increased secretion of platelet activators resulting in platelet hyperactivation [137].

**Figure 3:**
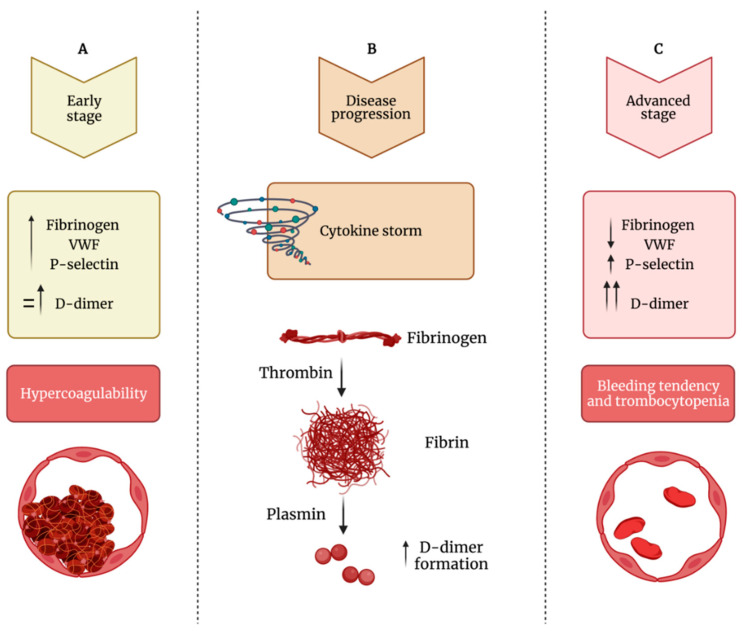
Impaired balance between hypercoagulability and bleeding tendency during COVID-19 progression. (**A**) During the early phase of the infection, patients show increased levels of fibrinogen, VWF (von Willebrand factor) and P-selectin and normal to mildly increased levels of D-dimer, leading to hypercoagulability. (**B**) During the disease’s progression, there is increased formation of D-dimer. (**C**) In the advanced stage of the infection, fibrinogen and VWF levels decrease, while D-dimer levels strongly increased; this phase is characterized by thrombocytopenia and bleeding diathesis.

Clinical observations may induce to hypothesize that in COVID-19 patients the measurement of D-dimer, fibrinogen, VWF, and the platelet activation marker P-selectin may help clinicians in deciding treatment strategy on the basis of correct clinical diagnosis [138]. Specifically, as shown in Figure 3, during early stages, COVID-19 patients show normal to slightly enhanced levels of D-dimer, fibrinogen, VWF and P-selectin, and platelet activation. If untreated, D-dimer rapidly increases and also fibrinogen, VWF and P-selectin further increase leading to platelet hyperactivation, clot formation, and thrombotic events. During the late stage of the disease, critically ill patients show cytokine storm, still high levels of P-selectin and D-dimer, while fibrinogen and VWF decrease because they are depleted by damaged endothelial cells or hyperactivated platelets that, at this stage, show thrombocytopenia (Figure 3).

## 7. Alterations of Platelet Indices in COVID-19

A common complication in patients affected by COVID-19 is thrombocytopenia, which indicates poor prognosis and high mortality of hospitalized COVID-19 patients [139,140]. A meta-analysis of 31 studies including 7613 patients found lower platelet count in severe COVID-19 infection associated with a three-fold increase in the risk of developing severe COVID-19 [141]. Indeed, lower platelet count was found in patients with either more severe illness or poor outcomes and in non-survivors. Curiously, thrombocytopenia has been reported to be not significantly related to intensive care unit (ICU) admission. This fact may be justified considering that thrombocytopenia tends to significantly appear in the late clinical stage of COVID-19 [142].

**Figure 4:**
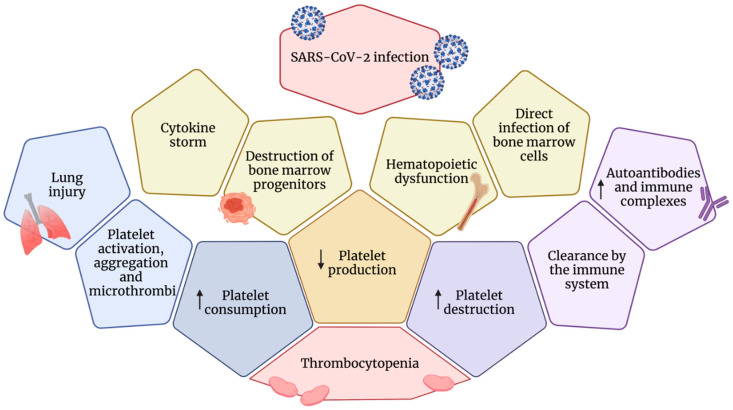
Mechanisms leading to thrombocytopenia in COVID-19 patients. Thrombocytopenia in SARS-CoV-2 is related to decreased platelet production, increased platelet consumption, and destruction.

The mechanisms proposed to explain this hematological abnormality are not definitely clarified. However, three aspects can be assumed to explain thrombocytopenia in SARS-CoV-2 basically related to decreased platelet production, increased platelet consumption, and disruption [143] (Figure 4).

Since coronaviruses can directly infect bone marrow cells, resulting in abnormal hematopoiesis [144], a possible cause of decreased platelet generation could be also the bone marrow suppression due to the SARS-CoV-2 binding to specific receptors leading to decreased primary platelet formation [144].

In addition, bone marrow suppression can be a consequence of the COVID-19-induced aberrant inflammatory cytokine production that causes immune damage to the lungs with the disruption of hematopoietic progenitor cells [145] with consequently decreased platelet primary production.

Actually, given that a large number of megakaryocytes release platelets during pulmonary circulation [23], one can also speculate that the presence of the ARDS secondary to COVID-19 pneumonia contributes to dysfunction of megakaryopoiesis, leading to a process of megakaryocytes rupture, blocking platelet release into pulmonary circulation and then reducing the number of systemic platelet circulation.

It is known that the increase in autoantibodies and immune complexes leads to platelet destruction, and this phenomenon has been frequently reported in patients with COVID-19 [146].

An immunological mechanism of thrombocytopenia independent of complement that leads to platelet disruption may be attributable to antibody anti-platelet causing platelet lysis via reactive oxygen species [147]. Indeed, this immunologic thrombocytopenia, a common complication in patients affected by human immunodeficiency virus (HIV)-1 disease [148], has been demonstrated to be associated with circulating immune complexes containing platelet membrane components (GPIIIa49-66) and anti-platelet membrane antibodies. Immune complexes and antibodies when deposited on platelet surfaces would be recognized by reticuloendothelial cells and destroyed resulting in massive platelet destruction.

SARS-CoV-2 causes thrombocytopenia also for increased platelet consumption. Indeed, damage of lung tissue following viral infection and inflammation triggers platelet activation and aggregation with formation of microthrombi, thus leading to platelet consumption [149]. One cause of depleted platelet number may be attributable to the increased secretion of coagulation biomarkers, such as D-dimer, P-selectin, fibrinogen, and VWF, that can induce platelet hyperactivation and aggregation after binding to platelet receptors. In this case, platelet counts may be lower because aggregated platelets are not counted by a platelet count analyzer.

Besides platelet counts, other indices of platelets, such as platelet size and maturity, have been shown to be associated with increased platelet activation and adverse clinical events [150,151,152]. Larger platelets are more active with a greater prothrombotic phenotype [153], and mean platelet volume (MPV) is a predictor of CV events being associated with increased morbidity and all-cause mortality [154]. In comparison with matched critically ill patients not affected by COVID-19, patients with COVID-19 showed larger MPV [155] and a tendency to have elevated immature platelet fraction (IPF), mirroring the number of circulating young platelets [155]. In comparison with mature platelets, immature platelets contained higher amounts of RNA [156] with prothrombotic transcriptomic profiles [157]. Patients deceased for COVID-19 had shown a more prothrombotic platelet profile due to the fact of SARS-CoV-2’s ability to induce transcriptomic changes to megakaryocytes [17]. Indeed, as noted by Barrett et al., the direct viral–megakaryocytes interaction was able to influence the expression of transcripts resulting in enrichment of genes involved in metabolic and oxidative pathways related not only to platelet degranulation but also to platelet count, size, and immaturity, all contributing to a hyperactive phenotype. In multiple clinical settings, increased values of IPF were associated with adverse CV outcomes and mortality [158,159,160]. Noteworthy was that COVID-19 positive patients showed IPF ≥ 8% at platelet count up to 251 × 10^9^/L, whereas in non-COVID-19 patients, a relative IPF ≥ 8% was observed only in individuals with platelet counts lower than 70 × 10^9^/L [155]. Taken together, these findings suggest that megakaryocytes were stimulated to produce large immature platelets in response to increased platelet consumption.

## 8. Vaccine-Induced Thrombocytopenia and Thrombosis

Before worldwide marketing and distribution, SARS-CoV-2 vaccines were tested for safety and effectiveness by clinical trials and pooled analyses. However, the adenoviral vector-based vaccine started to raise suspicions of atypical coagulopathies after that millions of doses were administrated. In particular, the vaccine Chimpanzee Adenovirus Oxford 1 (ChAdOx1) nCov-19, formulated by AstraZeneca, was reported to be responsible for the occurrence of arterial and venous thromboembolism mostly in under 60 year old women [161]. Although rare events, approximately 1/100,000 recipients, the European Medicines Agency (EMA) wanted to further examine those cases [162], and atypical thrombosis involving cerebral and splanchnic veins were reported [163]. In these patients, pulmonary emboli were also observed. Other disorders requiring medical intervention were the DIC and a severe thrombocytopenia generally occurring 5–30 days after vaccination [163,164].

Though the etiology has not yet been well clarified, the involvement of anti-PF4 antibodies considered to exert a key role in what was initially described as similar to HIT has been established, with the difference that patients with vaccine-induced immune thrombotic thrombocytopenia (VITT) have never received heparin. Indeed, VITT differs from HIT not only for the absence of prior exposure to heparin but also because anti-PF4 antibodies in VITT are oligoclonal instead of polyclonal and the binding sites to PF4 are different [165].

**Figure 5:**
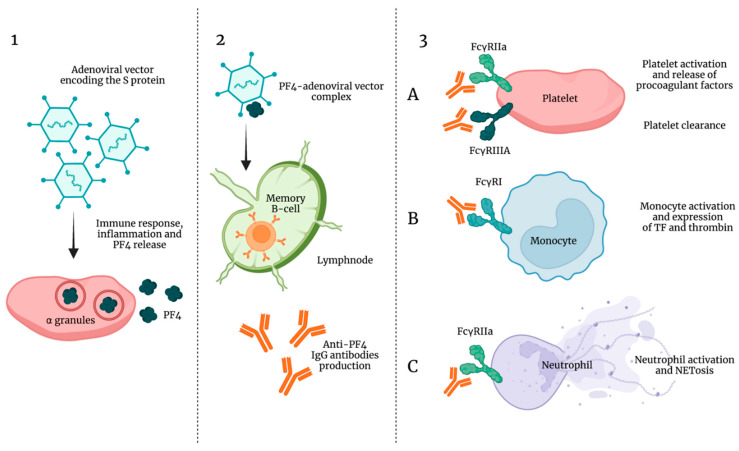
Pathogenesis of vaccine-induced thrombocytopenia and thrombosis (VITT). (**1**) Adenoviral vector encoding the S protein triggers an inflammatory response and platelet release of PF-4. (**2**) PF4–adenoviral vector complexes stimulate B-cells to produce anti-PF4 IgG antibodies (VITT antibodies). (**3**) (**A**) VITT antibodies bind FcγRIIa on the platelet surface causing platelet activation and release of procoagulant factors; VITT antibodies bind FcγRIIIA causing platelet clearance and thrombocytopenia. (**B**) VITT antibodies bind FcγRI on the monocyte surface causing monocyte activation and expression of TF and thrombin. (**C**) VITT antibodies bind FcγRIIa on the neutrophil surface causing neutrophil activation and NETosis.

A plausible explanation of VITT could be that adenoviral vector encoding the S protein triggers an immune response with a subsequent inflammatory response and platelet activation followed by PF-4 release, a cytokine that promotes coagulation via neutralization of heparin-like molecules on endothelial cells (Figure 5).

However, even though the SARS-CoV2 spike protein shows a similar epitope with PF4, it has been recently excluded that the SARS-CoV2 spike protein promotes the immune response responsible for VITT [166].

As already previously described, the complexes PF4–endothelial polyanionic proteoglycans stimulate extrafollicular B cells to produce IgG antibodies which exert positive feedback on platelet activation. From this point on, thrombogenesis and hemostasis disorders would resemble those induced by HIT autoantibodies, thus inducing a pan-cellular response combined with a Fcχ receptor-dependent stimulation of neutrophils and monocytes with the subsequent NETosis and TF and thrombin generation [167,168]. In addition, a role in the immune complex-mediated thrombotic sequelae may derive from complement activation which facilitates FcγR-independent monocyte TF expression, IgG binding to the cell surface FcγRs, and platelet adhesion to damaged endothelium [169].

Moreover, the incidence of VITT is very rare among adenoviral vectors and appears exceptional with mRNA-based vaccines [170].

## 9. Conclusions

Several independent studies are in line to consider platelets the frontline of COVID-19 pathogenesis for their involvement in different stages of SARS-CoV-2 infection. The most common complication in patients severely affected by COVID-19 is thrombocytopenia, basically related to decreased platelet production and increased platelet consumption and disruption.

However, platelet abnormalities in COVID-19 are not only quantitative. Indeed, it has been clearly established that platelets are also hyperreactive, thus consistently contributing to the overwhelming of thromboinflammatory events. Moreover, it cannot be excluded that platelets may also work as a reservoir for SARS-CoV-2 infection, replication, and spread. Indeed, further studies are needed to clarify these potential aspects of platelets versus the virion responsible for COVID-19. Additionally, not only ACE2 but also other platelet receptors have been reported to regulate SARS-CoV-2 engagement of platelets. In any case, platelets participate actively to intravascular thrombosis representing a link between host defense and thromboinflammation, a process in which their interactions with activated neutrophils, monocytes, coagulation systems and endothelial cells lead to intravascular clot formation from small to large vessels. Certainly, platelet potential to contribute to the overwhelming thromboinflammatory responses might suggest that a tailored antiplatelet therapy in addition to heparin could improve the outcomes during COVID-19. Further exploration of this pharmacological strategy needs to be done.

## References

[1] Ackermann M., Verleden S.E., Kuehnel M., Haverich A., Welte T., Laenger F., Vanstapel A., Werlein C., Stark H., Tzankov A. (2020). Pulmonary Vascular Endothelialitis, Thrombosis, and Angiogenesis in COVID-19. N. Engl. J. Med..

[2] Yeaman M.R. (2010). Bacterial-Platelet Interactions: Virulence Meets Host Defense. Future Microbiol..

[3] De Stoppelaar S.F., van’t Veer C., van der Poll T. (2014). The Role of Platelets in Sepsis. Thromb. Haemost..

[4] Jenne C.N., Kubes P. (2015). Platelets in Inflammation and Infection. Platelets.

[5] Mussbacher M., Salzmann M., Brostjan C., Hoesel B., Schoergenhofer C., Datler H., Hohensinner P., Basílio J., Petzelbauer P., Assinger A. (2019). Cell Type-Specific Roles of NF-ΚB Linking Inflammation and Thrombosis. Front. Immunol..

[6] Liverani E., Mondrinos M.J., Sun S., Kunapuli S.P., Kilpatrick L.E. (2018). Role of Protein Kinase C-Delta in Regulating Platelet Activation and Platelet-Leukocyte Interaction during Sepsis. PLoS ONE.

[7] Acanfora D., Acanfora C., Ciccone M.M., Scicchitano P., Bortone A.S., Uguccioni M., Casucci G. (2021). The Cross-Talk between Thrombosis and Inflammatory Storm in Acute and Long-COVID-19: Therapeutic Targets and Clinical Cases. Viruses.

[8] Cicala C., Cirino G. (1998). Linkage between Inflammation and Coagulation: An Update on the Molecular Basis of the Crosstalk. Life Sci..

[9] Esmon C.T., Xu J., Lupu F. (2011). Innate Immunity and Coagulation. J. Thromb. Haemost..

[10] Taus F., Salvagno G., Canè S., Fava C., Mazzaferri F., Carrara E., Petrova V., Barouni R.M., Dima F., Dalbeni A. (2020). Platelets Promote Thromboinflammation in SARS-CoV-2 Pneumonia. Arter. Thromb. Vasc. Biol..

[11] Hansson G.K., Robertson A.-K.L., Söderberg-Nauclér C. (2006). Inflammation and Atherosclerosis. Annu. Rev. Pathol..

[12] Stark K., Massberg S. (2021). Interplay between Inflammation and Thrombosis in Cardiovascular Pathology. Nat. Rev. Cardiol..

[13] McFadyen J.D., Stevens H., Peter K. (2020). The Emerging Threat of (Micro)Thrombosis in COVID-19 and Its Therapeutic Implications. Circ. Res..

[14] Melillo F., Napolano A., Loffi M., Regazzoni V., Boccellino A., Danzi G.B., Cappelletti A.M., Rovere-Querini P., Landoni G., Ingallina G. (2021). Myocardial Injury in Patients with SARS-CoV-2 Pneumonia: Pivotal Role of Inflammation in COVID-19. Eur. J. Clin. Investig..

[15] Attiq A., Yao L.J., Afzal S., Khan M.A. (2021). The Triumvirate of NF-ΚB, Inflammation and Cytokine Storm in COVID-19. Int. Immunopharmacol..

[16] Manne B.K., Denorme F., Middleton E.A., Portier I., Rowley J.W., Stubben C., Petrey A.C., Tolley N.D., Guo L., Cody M. (2020). Platelet Gene Expression and Function in Patients with COVID-19. Blood.

[17] Barrett T.J., Bilaloglu S., Cornwell M., Burgess H.M., Virginio V.W., Drenkova K., Ibrahim H., Yuriditsky E., Aphinyanaphongs Y., Lifshitz M. (2021). Platelets Contribute to Disease Severity in COVID-19. J. Thromb. Haemost..

[18] Zhang Y., Cao W., Jiang W., Xiao M., Li Y., Tang N., Liu Z., Yan X., Zhao Y., Li T. (2020). Profile of Natural Anticoagulant, Coagulant Factor and Anti-Phospholipid Antibody in Critically Ill COVID-19 Patients. J. Thromb. Thrombolysis.

[19] Zhou F., Yu T., Du R., Fan G., Liu Y., Liu Z., Xiang J., Wang Y., Song B., Gu X. (2020). Clinical Course and Risk Factors for Mortality of Adult Inpatients with COVID-19 in Wuhan, China: A Retrospective Cohort Study. Lancet.

[20] Comer S.P., Cullivan S., Szklanna P.B., Weiss L., Cullen S., Kelliher S., Smolenski A., Murphy C., Altaie H., Curran J. (2021). COVID-19 Induces a Hyperactive Phenotype in Circulating Platelets. PLoS Biol..

[21] Zaid Y., Puhm F., Allaeys I., Naya A., Oudghiri M., Khalki L., Limami Y., Zaid N., Sadki K., Ben E.l. (2020). Platelets Can Associate with SARS-Cov-2 RNA and Are Hyperactivated in COVID-19. Circ. Res..

[22] Machlus K.R., Italiano J.E. (2013). The Incredible Journey: From Megakaryocyte Development to Platelet Formation. J. Cell Biol..

[23] Lefrançais E., Ortiz-Muñoz G., Caudrillier A., Mallavia B., Liu F., Sayah D.M., Thornton E.E., Headley M.B., David T., Coughlin S.R. (2017). The Lung Is a Site of Platelet Biogenesis and a Reservoir for Haematopoietic Progenitors. Nature.

[24] Semple J.W., Italiano J.E., Freedman J. (2011). Platelets and the Immune Continuum. Nat. Rev. Immunol..

[25] Ghasemzadeh M., Hosseini E. (2013). Platelet-Leukocyte Crosstalk: Linking Proinflammatory Responses to Procoagulant State. Thromb. Res..

[26] Wang Y., Ouyang Y., Liu B., Ma X., Ding R. (2018). Platelet Activation and Antiplatelet Therapy in Sepsis: A Narrative Review. Thromb. Res..

[27] Eisen D.P., Reid D., McBryde E.S. (2012). Acetyl Salicylic Acid Usage and Mortality in Critically Ill Patients with the Systemic Inflammatory Response Syndrome and Sepsis. Crit. Care Med..

[28] Valerio-Rojas J.C., Jaffer I.J., Kor D.J., Gajic O., Cartin-Ceba R. (2013). Outcomes of Severe Sepsis and Septic Shock Patients on Chronic Antiplatelet Treatment: A Historical Cohort Study. Crit. Care Res. Pr..

[29] Li Z., Delaney M.K., O’Brien K.A., Du X. (2010). Signaling during Platelet Adhesion and Activation. Arter. Thromb. Vasc. Biol..

[30] Pretorius E. (2019). Platelets as Potent Signaling Entities in Type 2 Diabetes Mellitus. Trends Endocrinol. Metab..

[31] Estevez B., Shen B., Du X. (2015). Targeting Integrin and Integrin Signaling in Treating Thrombosis. Arter. Thromb. Vasc. Biol..

[32] Xu X.R., Carrim N., Neves M.A.D., McKeown T., Stratton T.W., Coelho R.M.P., Lei X., Chen P., Xu J., Dai X. (2016). Platelets and Platelet Adhesion Molecules: Novel Mechanisms of Thrombosis and Anti-Thrombotic Therapies. Thromb. J..

[33] Gao W., Shi P., Chen X., Zhang L., Liu J., Fan X., Luo X. (2018). Clathrin-Mediated Integrin AIIbβ3 Trafficking Controls Platelet Spreading. Platelets.

[34] Onselaer M.-B., Hardy A.T., Wilson C., Sanchez X., Babar A.K., Miller J.L.C., Watson C.N., Watson S.K., Bonna A., Philippou H. (2017). Fibrin and D-Dimer Bind to Monomeric GPVI. Blood Adv..

[35] Loyau S., Dumont B., Ollivier V., Boulaftali Y., Feldman L., Ajzenberg N., Jandrot-Perrus M. (2012). Platelet Glycoprotein VI Dimerization, an Active Process Inducing Receptor Competence, Is an Indicator of Platelet Reactivity. Arter. Thromb. Vasc. Biol..

[36] Slater A., Perrella G., Onselaer M.-B., Martin E.M., Gauer J.S., Xu R.-G., Heemskerk J.W., Ariëns R.A.S., Watson S.P. (2019). Does Fibrin(Ogen) Bind to Monomeric or Dimeric GPVI, or Not at All?. Platelets.

[37] Induruwa I., Moroi M., Bonna A., Malcor J.-D., Howes J.-M., Warburton E.A., Farndale R.W., Jung S.M. (2018). Platelet Collagen Receptor Glycoprotein VI-Dimer Recognizes Fibrinogen and Fibrin through Their D-Domains, Contributing to Platelet Adhesion and Activation during Thrombus Formation. J. Thromb. Haemost..

[38] Mammadova-Bach E., Ollivier V., Loyau S., Schaff M., Dumont B., Favier R., Freyburger G., Latger-Cannard V., Nieswandt B., Gachet C. (2015). Platelet Glycoprotein VI Binds to Polymerized Fibrin and Promotes Thrombin Generation. Blood.

[39] Jooss N.J., De Simone I., Provenzale I., Fernández D.I., Brouns S.L.N., Farndale R.W., Henskens Y.M.C., Kuijpers M.J.E., Ten Cate H., van der Meijden P.E.J. (2019). Role of Platelet Glycoprotein VI and Tyrosine Kinase Syk in Thrombus Formation on Collagen-Like Surfaces. Int. J. Mol. Sci..

[40] Mangin P.H., Onselaer M.-B., Receveur N., Le Lay N., Hardy A.T., Wilson C., Sanchez X., Loyau S., Dupuis A., Babar A.K. (2018). Immobilized Fibrinogen Activates Human Platelets through Glycoprotein VI. Haematologica.

[41] Varga-Szabo D., Pleines I., Nieswandt B. (2008). Cell Adhesion Mechanisms in Platelets. Arter. Thromb. Vasc. Biol..

[42] Srichai M.B., Zent R., Zent R., Pozzi A. (2010). Integrin Structure and Function. Cell-Extracellular Matrix Interactions in Cancer.

[43] Bongiovanni D., Klug M., Lazareva O., Weidlich S., Biasi M., Ursu S., Warth S., Buske C., Lukas M., Spinner C.D. (2021). SARS-CoV-2 Infection Is Associated with a pro-Thrombotic Platelet Phenotype. Cell Death Dis..

[44] Etulain J., Martinod K., Wong S.L., Cifuni S.M., Schattner M., Wagner D.D. (2015). P-Selectin Promotes Neutrophil Extracellular Trap Formation in Mice. Blood.

[45] Hottz E.D., Azevedo-Quintanilha I.G., Palhinha L., Teixeira L., Barreto E.A., Pão C.R.R., Righy C., Franco S., Souza T.M.L., Kurtz P. (2020). Platelet Activation and Platelet-Monocyte Aggregate Formation Trigger Tissue Factor Expression in Patients with Severe COVID-19. Blood.

[46] Rayes J., Bourne J.H., Brill A., Watson S.P. (2020). The Dual Role of Platelet-Innate Immune Cell Interactions in Thrombo-Inflammation. Res. Pr. Thromb. Haemost..

[47] Woollard K.J., Suhartoyo A., Harris E.E., Eisenhardt S.U., Jackson S.P., Peter K., Dart A.M., Hickey M.J., Chin-Dusting J.P.F. (2008). Pathophysiological Levels of Soluble P-Selectin Mediate Adhesion of Leukocytes to the Endothelium through Mac-1 Activation. Circ. Res..

[48] Chow J.H., Khanna A.K., Kethireddy S., Yamane D., Levine A., Jackson A.M., McCurdy M.T., Tabatabai A., Kumar G., Park P. (2021). Aspirin Use Is Associated with Decreased Mechanical Ventilation, Intensive Care Unit Admission, and In-Hospital Mortality in Hospitalized Patients with Coronavirus Disease 2019. Anesth. Analg..

[49] (2021). RECOVERY Collaborative Group Aspirin in Patients Admitted to Hospital with COVID-19 (RECOVERY): A Randomised, Controlled, Open-Label, Platform Trial. Lancet.

[50] Bonaventura A., Vecchié A., Dagna L., Martinod K., Dixon D.L., Van Tassell B.W., Dentali F., Montecucco F., Massberg S., Levi M. (2021). Endothelial Dysfunction and Immunothrombosis as Key Pathogenic Mechanisms in COVID-19. Nat. Rev. Immunol..

[51] Guo L., Rondina M.T. (2019). The Era of Thromboinflammation: Platelets Are Dynamic Sensors and Effector Cells During Infectious Diseases. Front. Immunol..

[52] Koupenova M., Clancy L., Corkrey H.A., Freedman J.E. (2018). Circulating Platelets as Mediators of Immunity, Inflammation, and Thrombosis. Circ. Res..

[53] Hidalgo A., Chang J., Jang J.-E., Peired A.J., Chiang E.Y., Frenette P.S. (2009). Heterotypic Interactions Enabled by Polarized Neutrophil Microdomains Mediate Thromboinflammatory Injury. Nat. Med..

[54] Hamburger S.A., McEver R.P. (1990). GMP-140 Mediates Adhesion of Stimulated Platelets to Neutrophils. Blood.

[55] Zarbock A., Singbartl K., Ley K. (2006). Complete Reversal of Acid-Induced Acute Lung Injury by Blocking of Platelet-Neutrophil Aggregation. J. Clin. Investig..

[56] Grommes J., Alard J.-E., Drechsler M., Wantha S., Mörgelin M., Kuebler W.M., Jacobs M., von Hundelshausen P., Markart P., Wygrecka M. (2012). Disruption of Platelet-Derived Chemokine Heteromers Prevents Neutrophil Extravasation in Acute Lung Injury. Am. J. Respir. Crit. Care Med..

[57] Blanks J.E., Moll T., Eytner R., Vestweber D. (1998). Stimulation of P-Selectin Glycoprotein Ligand-1 on Mouse Neutrophils Activates Beta 2-Integrin Mediated Cell Attachment to ICAM-1. Eur. J. Immunol..

[58] Hidari K.I., Weyrich A.S., Zimmerman G.A., McEver R.P. (1997). Engagement of P-Selectin Glycoprotein Ligand-1 Enhances Tyrosine Phosphorylation and Activates Mitogen-Activated Protein Kinases in Human Neutrophils. J. Biol. Chem..

[59] Gear A.R.L., Camerini D. (2003). Platelet Chemokines and Chemokine Receptors: Linking Hemostasis, Inflammation, and Host Defense. Microcirculation.

[60] Petersen F., Bock L., Flad H.D., Brandt E. (1999). Platelet Factor 4-Induced Neutrophil-Endothelial Cell Interaction: Involvement of Mechanisms and Functional Consequences Different from Those Elicited by Interleukin-8. Blood.

[61] Von Hundelshausen P., Koenen R.R., Sack M., Mause S.F., Adriaens W., Proudfoot A.E.I., Hackeng T.M., Weber C. (2005). Heterophilic Interactions of Platelet Factor 4 and RANTES Promote Monocyte Arrest on Endothelium. Blood.

[62] Rossaint J., Herter J.M., Van Aken H., Napirei M., Döring Y., Weber C., Soehnlein O., Zarbock A. (2014). Synchronized Integrin Engagement and Chemokine Activation Is Crucial in Neutrophil Extracellular Trap-Mediated Sterile Inflammation. Blood.

[63] Slungaard A. (2005). Platelet Factor 4: A Chemokine Enigma. Int. J. Biochem. Cell. Biol..

[64] Caudrillier A., Kessenbrock K., Gilliss B.M., Nguyen J.X., Marques M.B., Monestier M., Toy P., Werb Z., Looney M.R. (2012). Platelets Induce Neutrophil Extracellular Traps in Transfusion-Related Acute Lung Injury. J. Clin. Investig..

[65] Koenen R.R., von Hundelshausen P., Nesmelova I.V., Zernecke A., Liehn E.A., Sarabi A., Kramp B.K., Piccinini A.M., Paludan S.R., Kowalska M.A. (2009). Disrupting Functional Interactions between Platelet Chemokines Inhibits Atherosclerosis in Hyperlipidemic Mice. Nat. Med..

[66] Looney M.R., Nguyen J.X., Hu Y., Van Ziffle J.A., Lowell C.A., Matthay M.A. (2009). Platelet Depletion and Aspirin Treatment Protect Mice in a Two-Event Model of Transfusion-Related Acute Lung Injury. J. Clin. Investig..

[67] Singbartl K., Forlow S.B., Ley K. (2001). Platelet, but Not Endothelial, P-Selectin Is Critical for Neutrophil-Mediated Acute Postischemic Renal Failure. FASEB J..

[68] Doerschuk C.M. (2001). Mechanisms of Leukocyte Sequestration in Inflamed Lungs. Microcirculation.

[69] Lane D.A., Denton J., Flynn A.M., Thunberg L., Lindahl U. (1984). Anticoagulant Activities of Heparin Oligosaccharides and Their Neutralization by Platelet Factor 4. Biochem. J..

[70] Lane D.A., Pejler G., Flynn A.M., Thompson E.A., Lindahl U. (1986). Neutralization of Heparin-Related Saccharides by Histidine-Rich Glycoprotein and Platelet Factor 4. J. Biol. Chem..

[71] Schoen P., Lindhout T., Franssen J., Hemker H.C. (1991). Low Molecular Weight Heparin-Catalyzed Inactivation of Factor Xa and Thrombin by Antithrombin III—Effect of Platelet Factor 4. Thromb. Haemost..

[72] Fiore M.M., Kakkar V.V. (2003). Platelet Factor 4 Neutralizes Heparan Sulfate-Enhanced Antithrombin Inactivation of Factor Xa by Preventing Interaction(s) of Enzyme with Polysaccharide. Biochem. Biophys. Res. Commun..

[73] Levine S.P., Wohl H. (1976). Human Platelet Factor 4: Purification and Characterization by Affinity Chromatography. Purification of Human Platelet Factor 4. J. Biol. Chem..

[74] Huang S.S., Huang J.S., Deuel T.F. (1982). Proteoglycan Carrier of Human Platelet Factor 4. Isolation and Characterization. J. Biol. Chem..

[75] Barber A.J., Käser-Glanzmann R., Jakábová M., Lüscher E.F. (1972). Characterization of a Chondroitin 4 -Sulfate Proteoglycan Carrier for Heparin Neutralizing Activity (Platelet Factor 4) Released from Human Blood Platelets. Biochim. Biophys. Acta.

[76] Busch C., Dawes J., Pepper D.S., Wasteson A. (1980). Binding of Platelet Factor 4 to Cultured Human Umbilical Vein Endothelial Cells. Thromb. Res..

[77] Marcum J.A., McKenney J.B., Rosenberg R.D. (1984). Acceleration of Thrombin-Antithrombin Complex Formation in Rat Hindquarters via Heparinlike Molecules Bound to the Endothelium. J. Clin. Investig..

[78] Brandt S., Krauel K., Jaax M., Renné T., Helm C.A., Hammerschmidt S., Delcea M., Greinacher A. (2015). Polyphosphates Form Antigenic Complexes with Platelet Factor 4 (PF4) and Enhance PF4-Binding to Bacteria. Thromb. Haemost..

[79] Kreimann M., Brandt S., Krauel K., Block S., Helm C.A., Weitschies W., Greinacher A., Delcea M. (2014). Binding of Anti-Platelet Factor 4/Heparin Antibodies Depends on the Thermodynamics of Conformational Changes in Platelet Factor 4. Blood.

[80] Palankar R., Kohler T.P., Krauel K., Wesche J., Hammerschmidt S., Greinacher A. (2018). Platelets Kill Bacteria by Bridging Innate and Adaptive Immunity via Platelet Factor 4 and FcγRIIA. J. Thromb. Haemost..

[81] Krauel K., Pötschke C., Weber C., Kessler W., Fürll B., Ittermann T., Maier S., Hammerschmidt S., Bröker B.M., Greinacher A. (2011). Platelet Factor 4 Binds to Bacteria, [Corrected] Inducing Antibodies Cross-Reacting with the Major Antigen in Heparin-Induced Thrombocytopenia. Blood.

[82] Cox D., Kerrigan S.W., Watson S.P. (2011). Platelets and the Innate Immune System: Mechanisms of Bacterial-Induced Platelet Activation. J. Thromb. Haemost..

[83] Zhi H., Dai J., Liu J., Zhu J., Newman D.K., Gao C., Newman P.J. (2015). Platelet Activation and Thrombus Formation over IgG Immune Complexes Requires Integrin AIIbβ3 and Lyn Kinase. PLoS ONE.

[84] Boylan B., Gao C., Rathore V., Gill J.C., Newman D.K., Newman P.J. (2008). Identification of FcgammaRIIa as the ITAM-Bearing Receptor Mediating AlphaIIbbeta3 Outside-in Integrin Signaling in Human Platelets. Blood.

[85] Keane C., Petersen H., Reynolds K., Newman D.K., Cox D., Jenkinson H.F., Newman P.J., Kerrigan S.W. (2010). Mechanism of Outside-in {alpha}IIb{beta}3-Mediated Activation of Human Platelets by the Colonizing Bacterium, Streptococcus Gordonii. Arter. Thromb. Vasc. Biol..

[86] Warkentin T.E. (2019). High-Dose Intravenous Immunoglobulin for the Treatment and Prevention of Heparin-Induced Thrombocytopenia: A Review. Expert Rev. Hematol..

[87] Salter B.S., Weiner M.M., Trinh M.A., Heller J., Evans A.S., Adams D.H., Fischer G.W. (2016). Heparin-Induced Thrombocytopenia: A Comprehensive Clinical Review. J. Am. Coll. Cardiol..

[88] Keragala C.B., Draxler D.F., McQuilten Z.K., Medcalf R.L. (2018). Haemostasis and Innate Immunity—A Complementary Relationship: A Review of the Intricate Relationship between Coagulation and Complement Pathways. Br. J. Haematol..

[89] Peerschke E.I.B., Yin W., Grigg S.E., Ghebrehiwet B. (2006). Blood Platelets Activate the Classical Pathway of Human Complement. J. Thromb. Haemost..

[90] Deravi N., Ahsan E., Fathi M., Hosseini P., Yaghoobpoor S., Lotfi R., Pourbagheri-Sigaroodi A., Bashash D. (2021). Complement Inhibition: A Possible Therapeutic Approach in the Fight against COVID-19. Rev. Med. Virol..

[91] Nicolai L., Leunig A., Brambs S., Kaiser R., Weinberger T., Weigand M., Muenchhoff M., Hellmuth J.C., Ledderose S., Schulz H. (2020). Immunothrombotic Dysregulation in COVID-19 Pneumonia Is Associated with Respiratory Failure and Coagulopathy. Circulation.

[92] Ahmad F., Kannan M., Ansari A.W. (2021). Role of SARS-CoV-2 -Induced Cytokines and Growth Factors in Coagulopathy and Thromboembolism. Cytokine Growth Factor Rev..

[93] Kanduc D. (2021). From Anti-SARS-CoV-2 Immune Respons.se to the Cytokine Storm via Molecular Mimicry. Antibodies.

[94] Yeaman M.R. (2014). Platelets: At the Nexus of Antimicrobial Defence. Nat. Rev. Microbiol..

[95] Li G., Fan Y., Lai Y., Han T., Li Z., Zhou P., Pan P., Wang W., Hu D., Liu X. (2020). Coronavirus Infections and Immune Responses. J. Med. Virol..

[96] Bautista-Vargas M., Bonilla-Abadía F., Cañas C.A. (2020). Potential Role for Tissue Factor in the Pathogenesis of Hypercoagulability Associated with in COVID-19. J. Thromb. Thrombolysis.

[97] Hottz E.D., Quirino-Teixeira A.C., Merij L.B., Pinheiro M.B.M., Rozini S.V., Bozza F.A., Bozza P.T. (2021). Platelet-Leukocyte Interactions in the Pathogenesis of Viral Infections. Platelets.

[98] Yang Y., Tang H. (2016). Aberrant Coagulation Causes a Hyper-Inflammatory Response in Severe Influenza Pneumonia. Cell. Mol. Immunol..

[99] Qu R., Ling Y., Zhang Y.-H.-Z., Wei L.-Y., Chen X., Li X.-M., Liu X.-Y., Liu H.-M., Guo Z., Ren H. (2020). Platelet-to-Lymphocyte Ratio Is Associated with Prognosis in Patients with Coronavirus Disease-19. J. Med. Virol..

[100] Goeijenbier M., van Wissen M., van de Weg C., Jong E., Gerdes V.E.A., Meijers J.C.M., Brandjes D.P.M., van Gorp E.C.M. (2012). Review: Viral Infections and Mechanisms of Thrombosis and Bleeding. J. Med. Virol..

[101] Kwong J.C., Schwartz K.L., Campitelli M.A., Chung H., Crowcroft N.S., Karnauchow T., Katz K., Ko D.T., McGeer A.J., McNally D. (2018). Acute Myocardial Infarction after Laboratory-Confirmed Influenza Infection. N. Engl. J. Med..

[102] Umapathi T., Kor A.C., Venketasubramanian N., Lim C.C.T., Pang B.C., Yeo T.T., Lee C.C., Lim P.L., Ponnudurai K., Chuah K.L. (2004). Large Artery Ischaemic Stroke in Severe Acute Respiratory Syndrome (SARS). J. Neurol..

[103] Wong R.S.M., Wu A., To K.F., Lee N., Lam C.W.K., Wong C.K., Chan P.K.S., Ng M.H.L., Yu L.M., Hui D.S. (2003). Haematological Manifestations in Patients with Severe Acute Respiratory Syndrome: Retrospective Analysis. BMJ.

[104] Wang W., Xu Y., Gao R., Lu R., Han K., Wu G., Tan W. (2020). Detection of SARS-CoV-2 in Different Types of Clinical Specimens. JAMA.

[105] Campbell R.A., Boilard E., Rondina M.T. (2021). Is There a Role for the ACE2 Receptor in SARS-CoV-2 Interactions with Platelets?. J. Thromb. Haemost..

[106] Zhang S., Liu Y., Wang X., Yang L., Li H., Wang Y., Liu M., Zhao X., Xie Y., Yang Y. (2020). SARS-CoV-2 Binds Platelet ACE2 to Enhance Thrombosis in COVID-19. J. Hematol. Oncol..

[107] Huang C., Wang Y., Li X., Ren L., Zhao J., Hu Y., Zhang L., Fan G., Xu J., Gu X. (2020). Clinical Features of Patients Infected with 2019 Novel Coronavirus in Wuhan, China. Lancet.

[108] Koupenova M., Freedman J.E. (2015). Platelets: The Unsung Hero of the Immune Response. J. Thromb. Haemost..

[109] Ding Y., Wang H., Shen H., Li Z., Geng J., Han H., Cai J., Li X., Kang W., Weng D. (2003). The Clinical Pathology of Severe Acute Respiratory Syndrome (SARS): A Report from China. J. Pathol..

[110] Malha L., Mueller F.B., Pecker M.S., Mann S.J., August P., Feig P.U. (2020). COVID-19 and the Renin-Angiotensin System. Kidney Int. Rep..

[111] Harrison D.G., Guzik T.J., Lob H.E., Madhur M.S., Marvar P.J., Thabet S.R., Vinh A., Weyand C.M. (2011). Inflammation, Immunity, and Hypertension. Hypertension.

[112] Dmitrieva N.I., Burg M.B. (2015). Elevated Sodium and Dehydration Stimulate Inflammatory Signaling in Endothelial Cells and Promote Atherosclerosis. PLoS ONE.

[113] Nemerson Y. (1988). Tissue Factor and Hemostasis. Blood.

[114] Mackman N., Tilley R.E., Key N.S. (2007). Role of the Extrinsic Pathway of Blood Coagulation in Hemostasis and Thrombosis. Arter. Thromb. Vasc. Biol..

[115] Grover S.P., Mackman N. (2018). Tissue Factor: An Essential Mediator of Hemostasis and Trigger of Thrombosis. Arter. Thromb. Vasc. Biol..

[116] Mackman N. (1997). Regulation of the Tissue Factor Gene. Thromb. Haemost..

[117] Wang J., Mahmud S.A., Bitterman P.B., Huo Y., Slungaard A. (2007). Histone Deacetylase Inhibitors Suppress TF-KappaB-Dependent Agonist-Driven Tissue Factor Expression in Endothelial Cells and Monocytes. J. Biol. Chem..

[118] Li Y.-D., Ye B.-Q., Zheng S.-X., Wang J.-T., Wang J.-G., Chen M., Liu J.-G., Pei X.-H., Wang L.-J., Lin Z.-X. (2009). NF-KappaB Transcription Factor P50 Critically Regulates Tissue Factor in Deep Vein Thrombosis. J. Biol. Chem..

[119] Mackman N. (2009). The Many Faces of Tissue Factor. J. Thromb. Haemost..

[120] Rosell A., Havervall S., von Meijenfeldt F., Hisada Y., Aguilera K., Grover S.P., Lisman T., Mackman N., Thålin C. (2021). Patients With COVID-19 Have Elevated Levels of Circulating Extracellular Vesicle Tissue Factor Activity That Is Associated with Severity and Mortality-Brief Report. Arter. Thromb. Vasc. Biol..

[121] Subrahmanian S., Borczuk A., Salvatore S., Fung K.-M., Merrill J.T., Laurence J., Ahamed J. (2021). Tissue Factor Upregulation Is Associated with SARS-CoV-2 in the Lungs of COVID-19 Patients. J. Thromb. Haemost..

[122] Bury L., Camilloni B., Castronari R., Piselli E., Malvestiti M., Borghi M., KuchiBotla H., Falcinelli E., Petito E., Amato F. (2021). Search for SARS-CoV-2 RNA in Platelets from COVID-19 Patients. Platelets.

[123] Barrett T.J., Lee A.H., Xia Y., Lin L.H., Black M., Cotzia P., Hochman J., Berger J.S. (2020). Platelet and Vascular Biomarkers Associate with Thrombosis and Death in Coronavirus Disease. Circ. Res..

[124] Shen B., Yi X., Sun Y., Bi X., Du J., Zhang C., Quan S., Zhang F., Sun R., Qian L. (2020). Proteomic and Metabolomic Characterization of COVID-19 Patient Sera. Cell.

[125] Bergmann C.C., Silverman R.H. (2020). COVID-19: Coronavirus Replication, Pathogenesis, and Therapeutic Strategies. Clevel. Clin. J. Med..

[126] Grobbelaar L.M., Venter C., Vlok M., Ngoepe M., Laubscher G.J., Lourens P.J., Steenkamp J., Kell D.B., Pretorius E. (2021). SARS-CoV-2 Spike Protein S1 Induces Fibrin(Ogen) Resistant to Fibrinolysis: Implications for Microclot Formation in COVID-19. Biosci. Rep..

[127] Kawase M., Kataoka M., Shirato K., Matsuyama S. (2019). Biochemical Analysis of Coronavirus Spike Glycoprotein Conformational Intermediates during Membrane Fusion. J. Virol..

[128] Watanabe Y., Allen J.D., Wrapp D., McLellan J.S., Crispin M. (2020). Site-Specific Glycan Analysis of the SARS-CoV-2 Spike. Science.

[129] Flores-Alanis A., Sandner-Miranda L., Delgado G., Cravioto A., Morales-Espinosa R. (2020). The Receptor Binding Domain of SARS-CoV-2 Spike Protein Is the Result of an Ancestral Recombination between the Bat-CoV RaTG13 and the Pangolin-CoV MP789. BMC Res. Notes.

[130] Letarov A.V., Babenko V.V., Kulikov E.E. (2021). Free SARS-CoV-2 Spike Protein S1 Particles May Play a Role in the Pathogenesis of COVID-19 Infection. Biochemistry.

[131] Engelmann B., Massberg S. (2013). Thrombosis as an Intravascular Effector of Innate Immunity. Nat. Rev. Immunol..

[132] Weyrich A.S., Zimmerman G.A. (2013). Platelets in Lung Biology. Annu. Rev. Physiol..

[133] Morrell C.N., Aggrey A.A., Chapman L.M., Modjeski K.L. (2014). Emerging Roles for Platelets as Immune and Inflammatory Cells. Blood.

[134] Thachil J. (2020). What Do Monitoring Platelet Counts in COVID-19 Teach Us?. J. Thromb. Haemost..

[135] Goshua G., Pine A.B., Meizlish M.L., Chang C.-H., Zhang H., Bahel P., Baluha A., Bar N., Bona R.D., Burns A.J. (2020). Endotheliopathy in COVID-19-Associated Coagulopathy: Evidence from a Single-Centre, Cross-Sectional Study. Lancet Haematol..

[136] Conti P., Caraffa A., Gallenga C.E., Ross R., Kritas S.K., Frydas I., Younes A., Di Emidio P., Ronconi G., Toniato E. (2020). IL-1 Induces Throboxane-A2 (TxA2) in COVID-19 Causing Inflammation and Micro-Thrombi: Inhibitory Effect of the IL-1 Receptor Antagonist (IL-1Ra). J. Biol. Regul. Homeost. Agents.

[137] Derhaschnig U., Schweeger-Exeli I., Marsik C., Cardona F., Minuz P., Jilma B. (2010). Effects of Aspirin and NO-Aspirin (NCX 4016) on Platelet Function and Coagulation in Human Endotoxemia. Platelets.

[138] Grobler C., Maphumulo S.C., Grobbelaar L.M., Bredenkamp J.C., Laubscher G.J., Lourens P.J., Steenkamp J., Kell D.B., Pretorius E. (2020). COVID-19: The Rollercoaster of Fibrin(Ogen), D-Dimer, Von Willebrand Factor, P-Selectin and Their Interactions with Endothelial Cells, Platelets and Erythrocytes. Int. J. Mol. Sci..

[139] Maquet J., Lafaurie M., Sommet A., Moulis G. (2020). Thrombocytopenia Is Independently Associated with Poor Outcome in Patients Hospitalized for COVID-19. Br. J. Haematol.

[140] Yang X., Yang Q., Wang Y., Wu Y., Xu J., Yu Y., Shang Y. (2020). Thrombocytopenia and Its Association with Mortality in Patients with COVID-19. J. Thromb. Haemost..

[141] Jiang S.-Q., Huang Q.-F., Xie W.-M., Lv C., Quan X.-Q. (2020). The Association between Severe COVID-19 and Low Platelet Count: Evidence from 31 Observational Studies Involving 7613 Participants. Br. J. Haematol..

[142] Zong X., Gu Y., Yu H., Li Z., Wang Y. (2021). Thrombocytopenia Is Associated with COVID-19 Severity and Outcome: An Updated Meta-Analysis of 5637 Patients with Multiple Outcomes. Lab. Med..

[143] Xu P., Zhou Q., Xu J. (2020). Mechanism of Thrombocytopenia in COVID-19 Patients. Ann. Hematol..

[144] Yang M., Ng M.H.L., Li C.K. (2005). Thrombocytopenia in Patients with Severe Acute Respiratory Syndrome (Review). Hematology.

[145] Kucia M., Ratajczak J., Bujko K., Adamiak M., Ciechanowicz A., Chumak V., Brzezniakiewicz-Janus K., Ratajczak M.Z. (2021). An Evidence That SARS-Cov-2/COVID-19 Spike Protein (SP) Damages Hematopoietic Stem/Progenitor Cells in the Mechanism of Pyroptosis in Nlrp3 Inflammasome-Dependent Manner. Leukemia.

[146] Zhang Y., Zeng X., Jiao Y., Li Z., Liu Q., Ye J., Yang M. (2020). Mechanisms Involved in the Development of Thrombocytopenia in Patients with COVID-19. Thromb. Res..

[147] Nardi M., Tomlinson S., Greco M.A., Karpatkin S. (2001). Complement-Independent, Peroxide-Induced Antibody Lysis of Platelets in HIV-1-Related Immune Thrombocytopenia. Cell.

[148] Murphy M.F., Metcalfe P., Waters A.H., Carne C.A., Weller I.V., Linch D.C., Smith A. (1987). Incidence and Mechanism of Neutropenia and Thrombocytopenia in Patients with Human Immunodeficiency Virus Infection. Br. J. Haematol..

[149] Chen W., Li Z., Yang B., Wang P., Zhou Q., Zhang Z., Zhu J., Chen X., Yang P., Zhou H. (2020). Delayed-Phase Thrombocytopenia in Patients with Coronavirus Disease 2019 (COVID-19). Br. J. Haematol..

[150] Briggs C., Kunka S., Hart D., Oguni S., Machin S.J. (2004). Assessment of an Immature Platelet Fraction (IPF) in Peripheral Thrombocytopenia. Br. J. Haematol..

[151] Kickler T.S., Oguni S., Borowitz M.J. (2006). A Clinical Evaluation of High Fluorescent Platelet Fraction Percentage in Thrombocytopenia. Am. J. Clin. Pathol..

[152] Jung H., Jeon H.-K., Kim H.-J., Kim S.-H. (2010). Immature Platelet Fraction: Establishment of a Reference Interval and Diagnostic Measure for Thrombocytopenia. Korean J. Lab. Med..

[153] Shah B., Valdes V., Nardi M.A., Hu L., Schrem E., Berger J.S. (2014). Mean Platelet Volume Reproducibility and Association with Platelet Activity and Anti-Platelet Therapy. Platelets.

[154] Chu S.G., Becker R.C., Berger P.B., Bhatt D.L., Eikelboom J.W., Konkle B., Mohler E.R., Reilly M.P., Berger J.S. (2010). Mean Platelet Volume as a Predictor of Cardiovascular Risk: A Systematic Review and Meta-Analysis. J. Thromb. Haemost..

[155] Wool G.D., Miller J.L. (2021). The Impact of COVID-19 Disease on Platelets and Coagulation. Pathobiology.

[156] Guthikonda S., Alviar C.L., Vaduganathan M., Arikan M., Tellez A., DeLao T., Granada J.F., Dong J.-F., Kleiman N.S., Lev E.I. (2008). Role of Reticulated Platelets and Platelet Size Heterogeneity on Platelet Activity after Dual Antiplatelet Therapy with Aspirin and Clopidogrel in Patients with Stable Coronary Artery Disease. J. Am. Coll. Cardiol..

[157] Bongiovanni D., Santamaria G., Klug M., Santovito D., Felicetta A., Hristov M., von Scheidt M., Aslani M., Cibella J., Weber C. (2019). Transcriptome Analysis of Reticulated Platelets Reveals a Prothrombotic Profile. Thromb. Haemost..

[158] Ibrahim H., Schutt R.C., Hannawi B., DeLao T., Barker C.M., Kleiman N.S. (2014). Association of Immature Platelets with Adverse Cardiovascular Outcomes. J. Am. Coll. Cardiol..

[159] Muronoi T., Koyama K., Nunomiya S., Lefor A.K., Wada M., Koinuma T., Shima J., Suzukawa M. (2016). Immature Platelet Fraction Predicts Coagulopathy-Related Platelet Consumption and Mortality in Patients with Sepsis. Thromb. Res..

[160] Wu Q., Ren J., Hu D., Jiang P., Li G., Anjum N., Wang G., Gu G., Chen J., Wu X. (2015). An Elevated Percentage of Reticulated Platelet Is Associated with Increased Mortality in Septic Shock Patients. Medicine.

[161] Dimitrova E.K. Vaxzevria (Previously COVID-19 Vaccine AstraZeneca). https://www.ema.europa.eu/en/medicines/human/EPAR/vaxzevria-previously-COVID-19-vaccine-astrazeneca.

[162] SARS-CoV-2 Variants of Concern as of 18 November 2021. https://www.ecdc.europa.eu/en/COVID-19/variants-concern.

[163] Greinacher A., Thiele T., Warkentin T.E., Weisser K., Kyrle P.A., Eichinger S. (2021). Thrombotic Thrombocytopenia after ChAdOx1 NCov-19 Vaccination. N. Engl. J. Med..

[164] Schultz N.H., Sørvoll I.H., Michelsen A.E., Munthe L.A., Lund-Johansen F., Ahlen M.T., Wiedmann M., Aamodt A.-H., Skattør T.H., Tjønnfjord G.E. (2021). Thrombosis and Thrombocytopenia after ChAdOx1 NCoV-19 Vaccination. N. Engl. J. Med..

[165] Liu Y., Shao Z., Wang H. (2021). SARS-CoV-2 Vaccine-Induced Immune Thrombotic Thrombocytopenia. Thromb. Res..

[166] Greinacher A., Selleng K., Mayerle J., Palankar R., Wesche J., Reiche S., Aebischer A., Warkentin T.E., Muenchhoff M., Hellmuth J.C. (2021). Anti-Platelet Factor 4 Antibodies Causing VITT Do Not Cross-React with SARS-CoV-2 Spike Protein. Blood.

[167] Rollin J., Pouplard C., Gruel Y. (2016). Risk Factors for Heparin-Induced Thrombocytopenia: Focus on Fcγ Receptors. Thromb. Haemost..

[168] Perdomo J., Leung H.H.L., Ahmadi Z., Yan F., Chong J.J.H., Passam F.H., Chong B.H. (2019). Neutrophil Activation and NETosis Are the Major Drivers of Thrombosis in Heparin-Induced Thrombocytopenia. Nat. Commun..

[169] Khandelwal S., Barnes A., Rauova L., Sarkar A., Rux A.H., Yarovoi S.V., Zaitsev S.S., Lambris J.D., Myoung S.S., Johnson A. (2021). Complement Mediates Binding and Procoagulant Effects of Ultralarge HIT Immune Complexes. Blood.

[170] Pishko A.M., Cuker A. (2021). Thrombosis After Vaccination with Messenger RNA–1273: Is This Vaccine-Induced Thrombosis and Thrombocytopenia or Thrombosis With Thrombocytopenia Syndrome?. Ann. Intern. Med..

